# Survival and prognostic factors of progressive multifocal leukoencephalopathy in people living with HIV in modern ART era

**DOI:** 10.3389/fcimb.2023.1208155

**Published:** 2023-11-08

**Authors:** Rui Jiang, Zichen Song, Li Liu, Xue Mei, Jianjun Sun, Tangkai Qi, Zhenyan Wang, Wei Song, Yang Tang, Junyang Yang, Shuibao Xu, Bihe Zhao, Yinzhong Shen, Renfang Zhang, Jun Chen

**Affiliations:** ^1^ Department of Infection and Immunology, Shanghai Public Health Clinical Center, Fudan University, Shanghai, China; ^2^ Scientifc Research Center, Shanghai Public Health Clinical Center, Fudan University, Shanghai, China; ^3^ Department of Liver Intensive Care Unit, Shanghai Public Health Clinical Center, Fudan University, Shanghai, China

**Keywords:** HIV/AIDS, progressive multifocal leukoencephalopathy, survival rate, prognostic factor, polyoma virus, HIV/AIDS-related neurological disorders

## Abstract

**Background:**

The incidence of progressive multifocal leukoencephalopathy (PML) in people living with HIV (PLWH) is 2%-4%. Currently, there is no effective therapeutic strategy for the treatment of PML in PLWH, resulting in a mortality of up to 50%. This study aimed to identify risk factors of death and prognostic markers in people living with HIV with PML.

**Methods:**

A retrospective cohort study of AIDS-related PML individuals was conducted from January 1, 2015, to October 1, 2022, in Shanghai, China. PLWH who were diagnosed with PML for the first time were included. Kaplan-Meier curve and Cox regression were used to analyze the survival and its predictors. Levels of inflammatory markers and immune checkpoint inhibitors in blood and cerebrospinal fluid (CSF) were measured in the prestored samples using bead-based multiplex assay Indolamine 2,3-dioxygenase was determined using ELISA.

**Results:**

Twenty of 71 subjects had initiated antiretroviral therapy (ART) before PML onset and no patients discontinued ART during this period. In total, 34 patients (47.9%) had opportunistic infections (OIs), the median CD4+ T cell count was 73.0 (33.0-149.0) cells/μL. The estimated probability of survival at six months was 78% (95% confidential intervals [CIs]:0.63-0.85). OIs, low CD4+ T cell count were associated with lower estimated six-month survival (hazard ratio 8.01, 95% CIs: 1.80-35.00, P=0.006 and 5.01, 95% CIs:1.57-16.03, p=0.007). Indolamine 2,3-dioxygenase activity in CSF of non-survivors group were higher than survivors group (p<0.05).

**Conclusions:**

The survival rate of AIDS-related PML in the modern ART era was higher than the survival rate a decade ago. Low CD4+T cell count, OIs, were all associated with death of individuals with AIDS-related PML. The role of IDO in AIDS-related PML warrant further investigation.

## 1 Introduction

Progressive multifocal leukoencephalopathy (PML) is a demyelinating disease caused by the polymavirus JC (JC virus [JCV]), which occurs frequently in immunosuppressed individuals, especially in people living with HIV (PLWH) (Augusto et al., 2015; Joly et al., 2023). Over the past few decades, an increased incidence of PML has been linked to the AIDS epidemic and the rising use of immunosuppressive drugs (Grebenciucova and Berger, 2018). The incidence of PML increased from 0.026 per 100,000 individuals per year during 1988-2010 to 0.11 during 2011-2013 in Sweden (Iacobaeus et al., 2018).

Before the advent of antiretroviral therapy (ART), the incidence of PML in PLWH was 2%-4% and PML is recorded a mortality rate for 90% of PLWH (Dworkin et al., 1999; Lima et al., 2017). The survival rate of patients with AIDS-related PML has improved with the development of ART. A multicenter retrospective cohort study published in 2008 included 61 patients with AIDS-related PML found a six-month survival rate of 47.7% (Falcó et al., 2008). Patients with AIDS-related PML have such a low survival rate, yet there is currently no specific treatment for PML other than initiating or optimizing ART (Melliez et al., 2018).

Risk factors and predictors of mortality in patients with AIDS-related PML have to be identified to improve patient management and accelerate the development of new therapies. Nevertheless, researches with larger samples on immune checkpoints and cytokines in CSF and plasma from AIDS-related PML patients are still lacking (Cortese et al., 2019). In addition to CD4+T cell count, JC virus load, and HIV viral load, the association between prognosis in patients with PML and blocking PD-1 receptor has been demonstrated. It has been observed that blocking the PD-1 receptor enhances the JCV-specific T cell immune response in patients with PML (Tan et al., 2012). In PML patients, CD4+T cells secrete IFN-γ and IL-4, which may play a key role in JC virus clearance (Bernard-Valnet et al., 2021). Besides, upregulation of the immune checkpoint molecules such as PD-1 and CTLA-4 can lead to T cell exhaustion, reducing the immune response of their pathogens so that JC virus is more susceptible to infection of the central nervous system in PLWH (Cortese et al., 2021). Additionally, some researches showed that macrophage chemoattractant protein-1 levels in cerebrospinal fluid (CSF) and JCV-specific T-cell and IgG responses were correlated with containment of JC virus and prognosis of AIDS-related PML (Marzocchetti et al., 2005; Khanna et al., 2009).

Herein, we analyzed the clinical outcome and measured levels of immune checkpoints and inflammatory markers in plasma and CSF to determinate the survival and prognostic factors of PML in PLWH.

## 2 Materials and methods

### 2.1 Study population

This retrospective study was conducted in the Department of Infectious Diseases and Immunology of the Shanghai Public Health Clinical Center (SPHCC), Shanghai, China. Experiments were approved by the ethics committee of the SPHCC (Ethics approval number: 2021-S051-01) in strict conformity with the ethical guidelines of the Declaration of Helsinki. Written informed consent was obtained from all study participants.

All PLWH with progressive neurological systems or typical magnetic resonance imaging (MRI) findings and presented to SPHCC between January 1,2015 and October 1,2022 were included on initial review. CSF and plasma samples were collected and immediately stored at -80°C until analysis.

### 2.2 Study design

Clinical data of 71 AIDS-related PML individuals were collected. The following characteristics were collected from the hospital information system: gender, age, comorbidities, opportunistic infections (OIs), CD4+ T cell count, CD8+ T cell count, HIV viral load, CSF glucose, CSF chloride, CSF protein, ART regimen, JC-virus viral load, the time from the initiation of ART to PML onset, delay in PML diagnosis and clinical features.

The inclusion criteria were as follows: (1) HIV-1 infection confirmed by Western blotting; (2) patients had neuropathologic demonstration of the typical histopathologic triad (demyelination, bizarre astrocytes, and enlarged oligodendroglial nuclei) or the techniques to show the presence of JC virus; (3) patients had at least one PML clinical symptoms (motor deficits, cerebellar ataxia, speech disorders, cognitive disorders, visual disturbances, facial paralysis and seizures) or imaging manifestations (MRI shows high-signal lesions on T2-weighted images and fluid-attenuated inversion recovery images, low-signal lesions on T1-weighted images, and lesions in PML can occur almost anywhere in the brain, mostly in the frontal and parieto-occipital regions). The exclusion criteria were patients younger than 18 years.

The definitive diagnosis of PML requires positive brain biopsy or clinical and radiographic criteria consistent with demonstrating the presence of the JC virus in CSF. Patients who had clinical and radiological findings coupled with PML but for whom lumbar puncture and brain biopsy was not performed were categorized as possible PML cases (Berger et al., 2013). We defined deterioration as the occurrence of one or more symptoms progressing, improvement as one or more symptoms getting better, and stabilization as no change in clinical symptoms.

The immune reconstitution inflammatory syndrome (IRIS) diagnosis was defined as typical presentation or paradoxical rapid worsening of PML after initiation of HAART (within 90 days) together with a sharp decrease in plasma HIV-1 viral load and increase in CD4+ T cell count with exclusion of other diseases. Comorbidities were defined based on the Charlson Comorbidity Index (Quan et al., 2011), which includes 12 major diseases categories. Our comorbidity data included diabetes, hypertension, lymphoma, cerebrovascular diseases, chronic lung disease and liver diseases. The number of individuals with AIDS-related OIs other than PML was 34(47.9%). Baseline CD4+T cell count and HIV viral load were defined using the test results at admission or the closest record at admission. For the prognosis analysis, survival time was defined as the time from the beginning of definitive and diagnosis of PML to death, loss to follow-up, or the end of follow-up (October 1, 2022). Patients were followed up by telephone after discharge from the hospital. The outcome was all-cause mortality during the follow-up period. In our analysis, the patients lost to follow-up were treated as censored observations in accordance with standard survival analysis practices. Specifically, their follow-up times were right-censored at the last known follow-up date, meaning that we considered them at risk up to that point but did not have information on their outcomes beyond that time.

### 2.3 Measurement of levels of soluble immune checkpoints and inflammation markers in plasma and CSF samples

Bead-based multiplex assay was performed to detect inflammation cytokines/chemokines levels on plasma and CSF samples from different donors. We used the Human Inflammation Panel 1 (BioLegend, San Diego, CA, USA) to determine IL-1β, IFN-α2, IFN-γ, TNF-α, MCP-1 (CCL2), IL-6, IL-8 (CXCL8), IL-10, IL-12p70, IL-17A, IL-18, IL-23, and IL-33;and the Human Immune Checkpoint Panel 1 (10-plex) to determine sCD25 (IL-2Ra), 4-1BB, B7.2 (CD86), Free Active TGF-β1, CTLA-4, PD-L1, PD-1, Tim-3, LAG-3, and Galectin-9.Multiplex assays were done following the manufacturers’ instructions. Data were collected and analyzed using LEGENDplex software v8.0 (BioLegend, San Diego, CA, USA).

Kynurenine (Kyn) and Tryptophan (Trp) are quantitatively determined by ELISA (MyBioSource, San Diego, CA, USA), respectively. The Kyn : Trp (K:T) ratio were calculated to indicate the activity of the inducible enzyme indolamine 2,3-dioxygenase (IDO).

### 2.4 Statistical analysis

Statistical analysis was performed using R software, version4.2.0 (www.r-project.org), GraphPad Prism version 8.3.1 (GraphPad software, La Jolla, CA) and SPSS statistics 21.0 (IBM, Armonk, NY, USA). The Shapiro-Wilk test was used to test the normality of data distribution. Normally distributed data were reported as the means and standard deviation (mean ± SD). Non-normally distributed data were presented as the medians and interquartile range (IQR). Categorical variables were summarized with frequency counts and presented as a rate (%).

The χ^2^test, t test and Wilcoxon exact test were used to test for statistically significant differences. Kaplan-Meier survival analysis and log-rank test were used to identify risk factors that associated with outcome. Univariate Cox proportional hazards regression analysis and stepwise Cox proportional hazards regression models were used to estimate the predictors of mortality. The median period of follow-up and its interquartile range were calculated for the entire study cohort according to the reverse Kaplan-Meier method. We used the likelihood ratio test to examine the potential effect modification by comparing models with and without interaction term. P<0.05 indicated statistical significance.

## 3 Results

### 3.1 Clinical characteristics of the study population

A total of 71 patients were included. Forty-five (63.4%) patients were confirmed PML: JC virus DNA was detected in CSF in all cases and brain biopsy confirmed the diagnosis in one case. Of all the patients, 88.7% were male, and the median age was 35.0 (29.0-36.0) years. The median time to diagnosis was 30(21-90) days. In total, 34 patients (47.9%) had OIs, and 17 (23.9%) had comorbidities. In addition, the median CD4+ T cell count was 73.0 (33.0-149.0) cells/μL, and the median HIV viral load was 4.6(3.3-5.4) log10 copies/ml. Twenty (28.2%) patients had received ART before PML onset, and 12 patients of them had received it for more than 6 months (**Table 1**). All the patients continued or initiated ART in the study. Overall, 28 (39.4%) received an integrase inhibitor-based regimen, and 15(21.1%) received a non-nucleoside reverse transcriptase inhibitor-based regimen. HIV-PML presented mostly with motor deficits (35.2%), cerebellar ataxia (33.8%), speech disorders (21.1%), cognitive disorders (8.5%), visual disturbances (4.2%), facial paralysis (2.8%), and seizures (2.8%) (**Supplementary Table 1**). Among the 62 patients for whom outcome is detailed with a median follow-up of 19 (16-33) months, 14 patients (19.7%) improved, 31 patients (43.7%) stabilized, whereas 12 patients (28%) worsened (**Table 1**).

**Table 1:** Baseline characteristics of PLWH with PML.

Characteristics	n=71
Male, n (%)	63(88.7%)
Age(years)	35.0(29.0-36.0)
Confirmed PML (biopsy or PCR), n (%)	45(63.4%)
Delay in PML diagnosis	30(21-90)
OIs, n (%)	34(47.9%)
Bacterial pneumonia	9(26.5%)
Nontuberculous mycobacteria	7(20.6%)
Cytomegalovirus	6(17.7%)
Cryptococcosis	2(5.9%)
Digestive tract fungal infections	2(5.9%)
Pneumocystis pneumonia	2(5.9%)
Herpes zoster	2(5.9%)
Mycobacterium tuberculosis	2(5.9%)
Pulmonary aspergillosis	1(2.9%)
Fungal meningitis	1(2.9%)
Viral encephalomyelitis	1(2.9%)
Talaromyces marnefei	1(2.9%)
Comorbidity, n (%)	17(23.9%)
ART before PML onset	20(28.2%)
ART>6 months	12(60.0%)
ART<6 months	8(40.0%)
CD4 lymphocyte count(cells/μL)^a^	73.0(33.0-149.0)
No. patients with CD4 lymphocytes ≤ 50 cells/μL, n (%)	26(36.6%)
No. patients with CD4 lymphocytes>50 cells/μL, n (%)	43(60.6%)
CD8 lymphocyte count(cells/μL)	614.5(371.5 -907.0)
CSF glucose(mmol/L)^b^	3.2(2.8-3.5)
CSF chloride(mmol/L)^b^	122.0(119.1-124.0)
CSF protein(mg/L)^b^	471.3(295.0-745.3)
CSF leukocyte(10^6^/L)	2.0(1.0-5.0)
HIV-1 viral load(log10copies/ml)^c^	4.6(3.3-5.4)
JC-virus viral load(log10copies/ml)^d^	2.3(1.7-4.0)
ART regimens^e^	
NRTIs+ INIs	28(39.4%)
NRTIs+NNRTIs	15(21.1%)
NRTIs+PIs	11(15.5%)
NRTIs+PIs+INIs	11(15.5%)
IRIS, n (%)	14(19.7%)
Clinical outcome	
Improvement	14(19.7%)
Stabilization	31(43.7%)
Deterioration	2(2.8%)
Deceased	15(21.1%)
Lost to follow-up	9(12.7%)

^a^: CD4 lymphocyte count was missing for 2 patients ^b^: The levels of CSF glucose, CSF chloride, and CSF protein were not measured in 6 patients ^c^: HIV viral load was missing for 21 patients ^d^: JC-virus viral load was missing for 58 patients ^e^: Four participants’ ART regimens were not documented.

### 3.2 Survival analysis among people living with HIV after PML diagnosis

**Figure 1:**
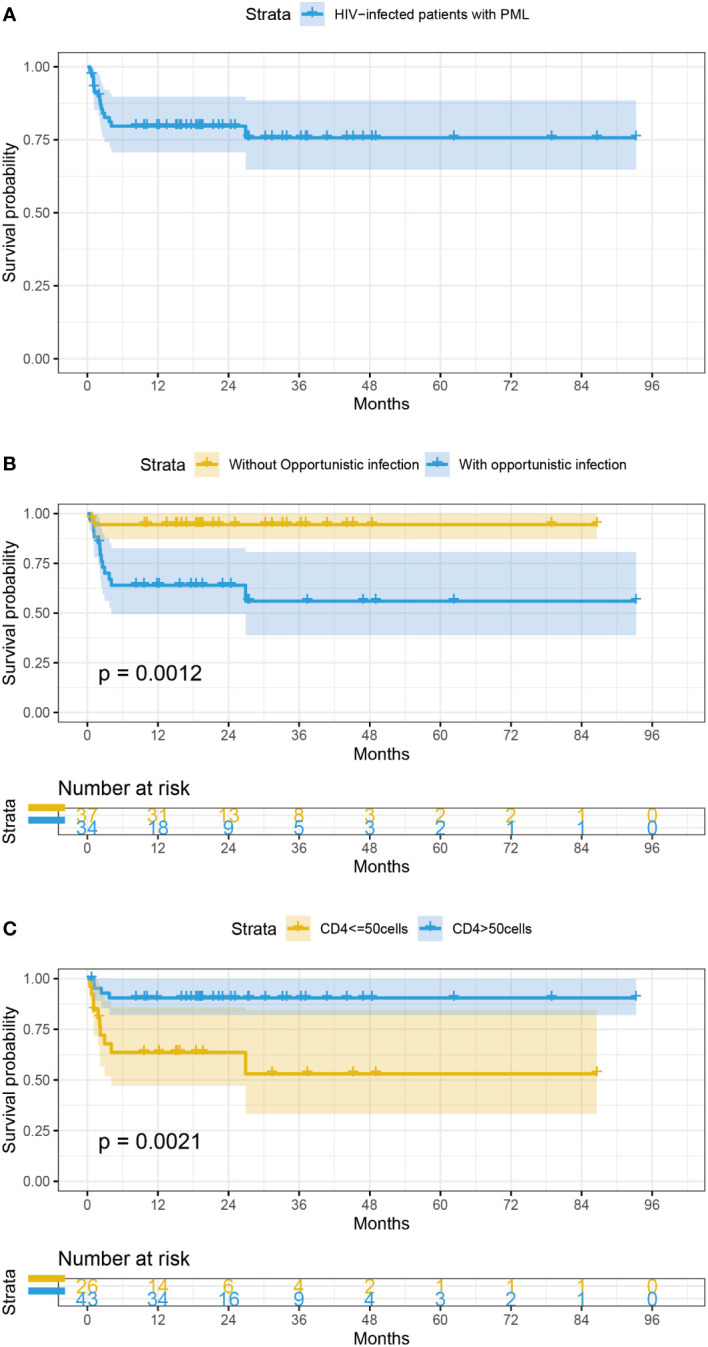
**(A)** Survival curves of PLWH with PML **(B)** Survival probability of AIDS-related PML patients categorized by OIs. **(C)** Survival probability of AIDS-related PML patients categorized by CD4+ T cell count at baseline.

After a median of 19 (16-33) months follow-up, 15 patients (21.1%) died despite receiving ART, and 9 patients (12.7%) were lost to follow-up. The lifetable method showed a survival rate of 78.0% [95% confidential intervals (CIs): 0.63-0.85]at 6 months,78.0% (95%CIs: 0.77-0.96) at 1 year,72% (95%CIs: 0.45-0.81) at 3 years (**Figure 1A**). All the causes of death were considered PML-related. Only OIs (HR = 8.01, 95% CIs: 1.80-35.00, P=0.006), and low CD4+ T cell count (HR=5.01, 95% CIs:1.57-16.03, p=0.007) were statistically significant for survival in univariate Cox regression analysis (**Table 2**). The survival of the subjects with different criteria (OIs and CD4+ T cell count) were presented using Kaplan-Meier curves (**Figures 1B, C**). Of note, the estimated 1-year survival was 89% in HIV patients with PML with CD4+ T cell count>50cell/μL at PML diagnosis compared to 59% in those with CD4+ T cell count ≤ 50cell/μL (**Figure 1C**). The estimated 1-year survival rate was 95.5% in patients without OIs and CD4+ T cell count >50 cells/μL, which showed optimal outcomes. In addition, IRIS is not a prognostic factor of survival of PLWH with PML (HR=1.15, 95% CIs 0.31-5.32, p =0.997) (**Table 2**).

**Table 2:** Hazard ratio of univariate analysis and multivariate analysis.

	Univariate analysis			Multivariate analysis		
	Hazard Ratio	95%CIs	P	Hazard Ratio	95%CIs	P
OIs^a^	8.01	1.80-35.00	0.006	6.10	1.35-27.55	0.019
CD4+ T cell count ≤ 50 cells/μL	5.01	1.57-16.03	0.007	3.11	1.05-9.23	0.041
CSF glucose ≤ 2.20mmol/L	2.02	0.45-9.04	0.356	–	–	0.175
Age	0.97	0.93-1.01	0.311	–	–	0.512
Comorbidity	2.60	0.74-9.30	0.132	–	–	0.555
IRIS	0.99	0.28-3.54	0.997	–	–	0.691
Confirmed PML	0.78	0.28-2.21	0.631	–	–	0.797
Delay in PML diagnosis	0.99	0.99-1.00	0.384			
Sex	0.64	0.08-4.94	0.672			
CSF chloride	0.99	0.96-1.10	0.780			
CSF protein	1.00	0.99-1.00^b^	0.871			
CSF leukocyte	1.01	0.99-1.03	0.440			
HIV-1 viral load	1.01	0.66-1.55	0.956			
JC-virus viral load	0.71	0.30-1.69	0.436			
ART before PML onset	1.10	0.34-3.40	0.900			

Age, IRIS, OIs, comorbidity, CD4+ T cell count ≤ 50 cells/μL, CSF glucose ≤ 2.20mmol/L were added to the model using stepwise procedures.

–: blank (In the multivariate analysis, SPSS 21.0 did not show 95% CIs and hazard ratio for variables that were not statistically significant).

^a^:P value for interaction was 0.960 between CD4+ T cell count ≤ 50 cells/μL and OIs ^b^: 95% CIs for hazard ratio of CSF protein: 0.998-1.001.

The variables including OIs, CD4+ T cell count, CSF glucose, IRIS, age, confirmed PML and comorbidity were included in a stepwise multivariate Cox proportional hazards model. There was no interaction between OIs and low CD4+ T cell count (p value for interaction =0.960) (**Table 2**). OIs [HR=6.01(1.35-27.55), p = 0.019] and CD4+ T cell count [HR=3.11(1.05-9.23), p = 0.041] were still associated with poor survival outcome.

### 3.3 Elevated levels of IDO activity in CSF of deceased patients

The levels of inflammatory markers and soluble immune checkpoint inhibitors were obtained in 31 plasma samples and 11 CSF samples at the diagnosis of PML. There was no significant difference in levels of immune checkpoint and inflammatory biomarkers in plasma between the survivors group and the non-survivors group (**Table 3**). In CSF, only IDO activity of the non-survivors group were significantly higher than that of the survivors group (p<0.05); (**Table 4**).

**Table 3:** Levels of soluble immune checkpoint inhibitors and inflammatory markers in plasma.

	Survivors group (n=13)	Non-survivors group(n=18)	P-value
	Median(range)	Median(range)	
CD86(pg/ml)	130.91(51.38-1944.66)	120.37(30.52-469.12)	0.761
CTLA-4(pg/ml)	5.28(2.46-44.68)	4.51(1.30-20.46)	0.403
Free active TGF-β1(pg/ml)	6.15(0.00-194.18)	14.74(0.00-37.95)	0.317
LAG-3(ng/ml)	3.73(0.71-70.11)	5.20(1.28-9.90)	0.761
PD-1(pg/ml)	41.61(9.78-323.84)	36.18(15.15-64.61)	0.279
PD-L1(pg/ml)	25.84(11.36-1178.12)	40.40(6.96-81.98)	0.227
Tim-3(ng/ml)	3.20 (5.92-14.11)	3.65 (1.21-65.09)	0.476
4-1BB (pg/ml)	101.62(27.19-1889.53)	101.74(60.84-208.74)	0.792
IFN-γ(pg/ml)	16.54(5.75-440.83)	15.29(6.96-46.79)	0.855
IFN-α(pg/ml)	5.32(2.25-23.24)	6.00(3.07-8.72)	0.555
IL-1β(pg/ml)	4.64(0.24-35.50)	7.72(0.32-619.69)	1
IL-6(pg/ml)	3.76(0.00-87.21)	11.58(0.00-20.11)	0.670
IL-8(pg/ml)	12.22(4.95-87.91)	12.88(3.16-39.39)	0.984
IL-10(pg/ml)	19.94(12.29-1554.03)	35.40(12.06-73.52)	0.502
IL-17A(pg/ml)	13.93(2.54-22.56)	11.93(6.80-14.67)	0.063
IL-18(ng/ml)	1.05(0.21-5.52)	1.08(0.11-2.04)	1
IL-23(pg/ml)	268.05(48.82-5291.38)	347.54(32.86-635.37)	1
MCP-1(pg/ml)	82.70(38.98-940.59)	71.39(18.29-238.78)	0.67

	Mean(SD)	Mean(SD)	P-value
IDO	0.05(0.02)	0.06(0.03)	0.361
IL-33(ng/ml)	8.80(4.90)	11.61(12.51)	0.487
Galectin-9(ng/ml)	34.26(19.76)	50.82(47.33)	0.180
IL-2Ra(ng/ml)	1.53(0.91)	2.16(1.53)	0.160
IL-12p70(pg/ml)	ND^a^	ND	ND
TNF-α(pg/ml)	ND	ND	ND

^a^: ND, no detectable.

**Table 4:** Levels of soluble immuecheckpoint inhibitors and inflammatory markers in cerebrospinal fluid (CSF).

	Survivors group (n=6)	Non-survivors group(n=5)	P-value
	Mean(SD)	Mean(SD)	
IDO	0.04(0.03)	0.07(0.02)	**0.048**
CD86(pg/ml)	44.14(13.42)	107.81(78.62)	0.145
CTLA-4(pg/ml)	2.22(1.02)	4.54(1.03)	0.216
Free active TGF-β1(pg/ml)	18.11(8.37)	27.02(20.37)	0.141
Galectin-9(ng/ml)	12.89 (1.409.05)	33.95 (33.86)	0.271
IL-2Ra(pg/ml)	76.03(118.64)	124.87(106.01)	0.494
LAG-3(ng/ml)	1.17 (0.59)	2.27(3.05)	0.434
PD-1(pg/ml)	19.32(9.53)	27.86(14.75)	0.274
PD-L1(pg/ml)	10.20(6.19)	26.35(21.06)	0.165
Tim-3(ng/ml)	9.27 (3.83)	14.10 (1.78)	0.581
4-1BB (pg/ml)	61.71(26.26)	150.07(127.81)	0.129
IFN-γ(pg/ml)	25.73(0.67)	5.57(0.62)	0.721
IFN-α(pg/ml)	2.14(0.44)	2.58(1.26)	0.439
IL-1β(pg/ml)	0.40(0.19)	0.40(0.17)	0.935
IL-6(pg/ml)	11.75(10.19)	9.40(7.21)	0.432
IL-8(pg/ml)	4.56(3.94)	7.90(6.25)	0.307
IL-10(pg/ml)	24.88(22.88)	22.01(17.99)	0.826
IL-17A(pg/ml)	5.71(4.46)	7.43(5.67)	0.587
IL-18(ng/ml)	2.83(1.47)	2.84(2.24)	0.994
IL-23(pg/ml)	112.01(159.56)	181.05(250.95)	0.592
IL-33(ng/ml)	3.34(7.99)	2.76(0.64)	0.224
MCP-1(pg/ml)	35.23(30.29)	42.39(44.71)	0.759
IL-12p70(pg/ml)	ND^a^	ND	ND
TNF-α(pg/ml)	ND	ND	ND

^a^: ND, no detectable. The bold value means P<0.05.

## 4 Discussion

The introduction of ART two decades ago greatly revolutionized treatment and allowed the reversion of HIV induced immunosuppression, increasing PML one-year survival from 10% to almost 50% (Lima et al., 2017). Our study showed that the overall survival rate of PLWH with PML was 78% at six months which is higher than previous data from other studies ten years ago and consistent with 72% of six-month survival rate from a study conducted in 2022 (Gasnault et al., 1999; Falcó et al., 2008; Marzocchetti et al., 2009; Anand et al., 2019; Joly et al., 2023),despite the fact that we had lower median CD4 T cell count than other studies. The improved survival outcome can result from the advancement of ART regimens and improvements in maintaining long-term adherence in recent years (Menéndez-Arias and Delgado, 2022).

We found higher mortality in patients with CD4+ T cell count ≤50 cells/μL compared with those with higher CD4+ T cell count, which was consistent with the previous reports (Berenguer et al., 2003; Marzocchetti et al., 2005). Therefore, the development of clinical grade T cell functional tests and laboratory tests for T cell subset analysis would contribute to more accurately predict and assess the prognosis of PML patients (Pavlovic et al., 2018). Interestingly, case reports have showed interleukin -7(IL-7) which have a role in boost CD4 T cell count could improve the outcome of patients with PML (Sospedra et al., 2014; Lajaunie et al., 2022). In this non-controlled retrospective study, survival did not differ from that expected in HIV/AIDS patients, but might have been improved in those with hematological malignancies, primary immunodeficiencies and transplant recipients. This may be explained by the fact that HIV-infected patients are at higher risk of developing problems related to immune reconstitution inflammatory response syndrome. The role of strategies that may boost CD4 T cell count needs further exploration.

The three most commonly HIV-related OIs were bacterial pneumonia, cytomegalovirus and nontuberculous mycobacteria among the study participants. A systematic review estimated that during the first year of ART, the incidences of all OIs declined to <2% in low- and middle-Income countries, except for unspecified tuberculosis, pneumonia tuberculosis, herpes zoster, and oral candidiasis, which remained the most common OIs (Low et al., 2016). However, OIs continue to cause significant morbidity and mortality among HIV patients despite receiving ART (UNAIDS, 2014). Of note, subjects without OIs and CD4+ T cell count >50 cells/μL showed optimal outcomes, which emphasizes the value of early HIV-infection diagnosis and ART initiation.

In recent years, PD-1 inhibitors have been reported to be effective in PML patients in some cases (Cortese et al., 2019; Rauer et al., 2019). However, pre-treatment stratification regarding treatment response rates is lacking. Therefore, a reliable strategy for selecting appropriate patients must be developed before larger studies can be conducted to further evaluate the efficacy of anti-PD-1 antibodies in patients with PML. To the best of our knowledge, this is the first study to determine the levels of immune checkpoints in plasma and CSF in PML patients. AIDS-related PML patients showed a higher percentage of PD-1 expression on CD4+ and CD8+ lymphocytes in blood and CSF than healthy controls (Tan et al., 2012). Thus, the use of PD-1 inhibitors can reduce JC viral load and increases CD4+ and CD8+ lymphocytes activity against the JC virus in PML patients. However, in our research the soluble form of PD-1 and other immune checkpoints showed no significant difference between the survivors and the non-survivors groups. This may be partially due to the fact that the enrolled patients were all newly diagnosed in our cohort, whereas only patients who did not show clinical improvement received the PD-1 inhibitors in other studies (Harel et al., 2018; Berzero et al., 2021).

We found that only IDO activity in CSF of non-survivor group in CSF was slightly higher than in survivors group among the measured immune checkpoints and inflammatory markers. IDO is produced by dendritic cells and regulatory T cells and affects T cell differentiation toward regulatory T cells and away from helper cells (Th-17) (Huang et al., 2020: Baer et al., 2021). Previous researches showed that increased plasma IDO activity is associated with HIV disease progression and mortality in PLWH (Pertovaara et al., 2006; Chen et al., 2014; Jenabian et al., 2015). Further evidence that the Kyn pathway is involved in bacterial meningitis came from a study that discovered higher levels of Kyn, IDO activity, and cytokines in the CSF of patients with bacterial meningitis (Coutinho et al., 2014). An observational cohort study confirmed the association between low CSF Trp and patient survival in tuberculous meningitis (van Laarhoven et al., 2018). Therefore, the IDO activity is also possible to be a prognostic marker of survival outcome of PML in PLWH. However, our result should be explained with caution due to the small samples size. Thus, the role of Kyn pathway and Trp metabolism in the pathogenesis of AIDS-related PML is worth further exploring.

There are also some limitations in our study including its retrospective nature. We have missing data for JC virus and HIV viral load, which previous studies (Gasnault et al., 2003; Marzocchetti et al., 2005) have shown to be important risk factors for death in PLWH with PML. Additionally, limitations of our study include the fact that some patients lacked a conclusive diagnosis and the small sample size of CSF and plasma. A non-significant interaction between low CD4+ T cell counts and occurrence of OIs is particularly related to the small sample size of the study. Future studies in a larger cohort are needed to validate our findings.

## 5 Conclusion

The survival rate of AIDS-related PML in the modern ART era was higher than it was a decade ago. Low CD4+T cell count, OIs, were all associated with death of individuals with AIDS-related PML. The role of IDO in AIDS-related PML warrant further investigation.

## Data availability statement

The raw data supporting the conclusions of this article will be made available by the authors, without undue reservation.

## Ethics statement

This study has been approved by Shanghai Public Health Clinical Center in China and informed consent was obtained from the patient involved. The approval number is 2021-S051-01. The studies were conducted in accordance with the local legislation and institutional requirements. The participants provided their written informed consent to participate in this study.

## Author contributions

JC and RZ conducted the study conception and design. RJ collected the data of patients. RJ and JC analyzed and interpreted the data. RJ wrote the manuscript. ZS and RJ contributed to the experiments. JC critically revised and finally approved the manuscript. RZ, XM TQ, JS, ZW, WS, YT,JY,SX and BZ supervised the project. All authors contributed to the article and approved the submitted version.
