# Supplementary material for: Survival and prognostic factors of progressive multifocal leukoencephalopathy in people living with HIV in modern ART era
Source: Front Cell Infect Microbiol. 2023 Nov 8;13:1208155. doi: 10.3389/fcimb.2023.1208155 (PMC10663249; doi:10.3389/fcimb.2023.1208155)
Supplement: Supplementary file 1 [file Table_1.docx]

| **Characteristics**   **n=71** | |
| --- | --- |
| **Clinical features** |  |
| Motor deficit | 25(35.2%) |
| Cerebellar ataxia | 24(33.8%) |
| Speech disorders | 15(21.1%) |
| Cognitive disorders | 6(8.5%) |
| Seizure | 3(4.2%) |
| Visual disturbances | 2(2.8%) |
| Facial paralysis | 2(2.8%) |

**Supplementary Table 1** Clinical features of the 71 included PML patients
